# Fast Functional Rehabilitation Protocol versus Plaster Cast Immobilization Protocol after Achilles Tendon Tenorrhaphy: Is It Different? Clinical, Ultrasonographic, and Elastographic Comparison

**DOI:** 10.3390/diagnostics12081824

**Published:** 2022-07-29

**Authors:** Mario Mosconi, Gianluigi Pasta, Salvatore Annunziata, Viviana Guerrieri, Matteo Ghiara, Simone Perelli, Camilla Torriani, Federico Alberto Grassi, Eugenio Jannelli

**Affiliations:** 1 Orthopedics and Traumatology Clinic, IRCCS Policlinico San Matteo Foundation, 27100 Pavia, Italy; mario.mosconi@unipv.it (M.M.); gianluigipasta@yahoo.it (G.P.); viviana.guerrieri@gmail.com (V.G.); m.ghiara@smatteo.pv.it (M.G.); c.torriani2@campus.unimib.it (C.T.); f.grassi@smatteo.pv.it (F.A.G.); eugenio.jannelli@libero.it (E.J.); 2 Specialization School in Orthopaedics and Traumatology, University of Pavia, 27100 Pavia, Italy; 3 Institut CAtalá de Traumatologia i Medicina de l’Esport (ICATME)—Hospital Universitari Dexeus, Universitat Autonoma de Barcelona, 08028 Barcelona, Spain; perelli.simone@gmail.com; 4 Department of Surgery and Morphologic Science, Orthopaedic Surgery Service, Hospital Del Mar, Universitat Autonoma de Barcelona, 08003 Barcelona, Spain; 5 Department of Medical and Surgical Specialties and Dentistry, University of Campania “Luigi Vanvitelli”, 80138 Naples, Italy

**Keywords:** Achilles tendon, early rehabilitation, ultrasonography, elastosonography, rupture, surgical repair

## Abstract

Background: the incidence of Achilles tendon (AT) rupture is rising; however, there is no clear consensus regarding the optimal treatment. The aim of this retrospective study was to compare instrumental and patient-reported outcome scores after fast functional rehabilitation (group A) versus plaster cast immobilization (group B) programs in patients who underwent AT tenorrhaphy. Methods: 33 patients, with similar clinical and demographic features, underwent open AT tenorrhaphy between January and July 2018. Of these, 15 patients were treated with fast functional rehabilitation program (group A), and 18 patients were treated with plaster cast immobilization protocol (group B). Sural triceps hypotrophy and functional scores (American Orthopaedic Foot and Ankle Society (AOFAS) Ankle–Hindfoot Score, and Achilles tendon Total Rupture Score (ATRS)) were recorded at a 12-month follow-up. Ultrasonography (US) and elastosonography (ES) were used to compare the characteristics of the tendons after surgery. Results: At 12 months, no significant differences in any of the patient-reported outcomes or the instrumental measurement tests were seen between the two groups. Conclusions: fast functional rehabilitation after AT surgical repair is safe, effective, and may be the first choice of treatment, especially in young, collaborative, and active patients.

## 1. Introduction

Achilles tendon (AT) rupture is one of the most frequent injuries of tendons [1]; its etiology is multifactorial, and its true nature remains unclear [2].

The overall incidence of AT rupture has increased recently because of the aging of the population, the growing prevalence of obesity, and the increased participation in recreational sports [3].

AT rupture used to occur at a mean age of 40 years [4], with a bimodal age distribution in which the first peak occurred in patients between 25 years and 40 years and the second peak in those over 60 years [3]. Men are 2 to 12 times more prone to AT rupture than women [2]; however, in a recent study, it has been demonstrated that age and sex do not have as important an influence on AT ruptures as they did in the past due to increased participation of the elderly and female populations in sports. In particular, an increase in neovascularization of the suffering tendon was found in young female athletes, due to functional overload. In this regard, therapy with extracorporeal shock waves has proved to be a useful strategy to “reset” the vascularization of the hyperperfused tendon, setting it to lower oximetry values in line with the “healthy” tendon portion [5,6]. The rupture of the AT is more frequent in patients who occasionally perform physical activity, due to an inadequate preparation for the physical effort. Ruptures are usually monolateral [7]. Bilateral ruptures are rare, and they are generally due to high-altitude falls [8].

Currently, there is no clear consensus on the optimal treatment of acute AT rupture, and conservative or surgical treatments are both accepted [9,10,11]. Conservative treatment is considered the best solution for elderly patients with comorbid medical conditions. Surgical treatment is performed on the vast majority of patients [8].

There are several techniques for AT repair, all of which involve the reapproximation of the torn ends of the tendon. The indications are based on different factors: the nature of the lesion (acute, subacute, or chronic lesion), the patient’s age, the type of activity performed by the patient, the patient’s comorbidities, the surgeon’s experience, and the economic implications (minimally invasive surgery is more expensive than open surgery, but both surgeries are more expensive than conservative treatment) [8,12,13].

The goals of surgical treatment are the prevention of secondary complications and recurrent lesions, early restoration of articular range of motion (ROM), and rapid recovery of strength and proprioception. Furthermore, the indications of rehabilitation protocol after surgery are controversial in the literature [14]. Postoperative rehabilitation must balance the risk of complications resulting from prolonged immobilization (e.g., adhesions and muscle atrophy) with the risk of tendon re-rupture due to early mobilization and tendon loading [15]. Surgical management of acute AT rupture has traditionally involved operative repair followed by a prolonged period of ankle immobilization in a rigid cast [1,9]. However, several recent studies found that functional bracing produced favorable outcomes in terms of motor performance, anthropometrics, and patient satisfaction [15].

This study aimed to compare the clinical, ultrasonographic, and elastographic effects of a “plaster cast” rehabilitation protocol (group B) versus a “fast functional” rehabilitation protocol (group A) after AT tenorrhaphy.

## 2. Materials and Methods

Thirty-three patients underwent open AT tenorrhaphy at our institution between January 2018 and July 2018. All surgeries were performed by the same senior surgeon.

### 2.1. Patient Selection

All patients were selected using a consecutive sampling technique. The inclusion criteria were: patients under 65 years old; patients with a primary lesion without previous physical or surgical therapy; patients with a medial third lesion; patients who underwent surgery within 10 days after injury. We excluded the following sets of patients: patients older than 65 years old, or who had a recurrent lesion following previous physical or surgical therapy; patients with medical conditions associated with higher risk of postoperative complications, such as rheumatic disease or collagenopathies; patients with a third proximal or distal lesion; patients with delayed surgery more than 10 days after injury (Table 1).

Both the fast functional rehabilitation (group A) and the plaster cast rehabilitation protocol (group B) were proposed to all the patients we operated on.

### 2.2. Preoperative Assessment

**Figure 1:**
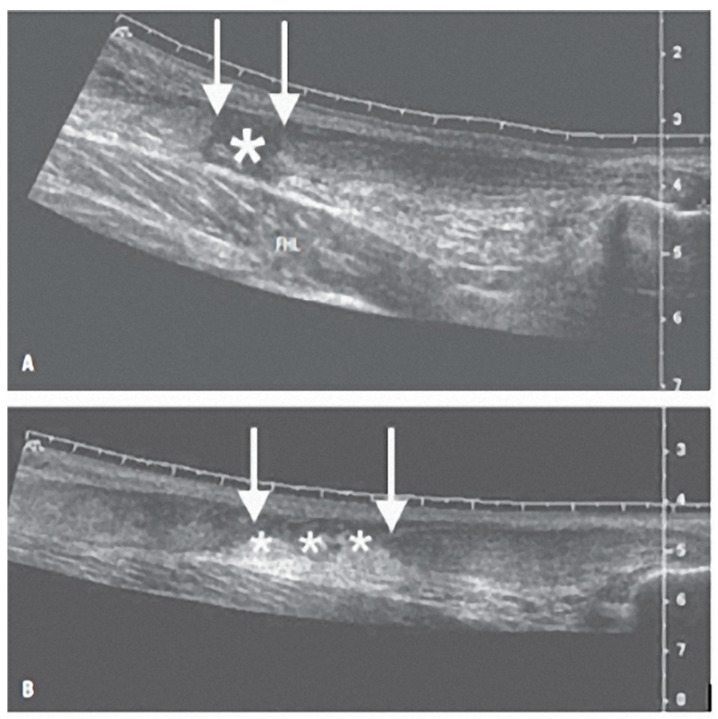
(**A**) Longitudinal scan image of complete Achilles tendon rupture; gap in the tendon (*) and the ends of the torn tendon (below the arrows). The muscular belly of the flexor hallucis longus muscle (FHL) is clearly visible below the lesion. (**B**) Image of longitudinal scan, complete rupture of the Achilles tendon. Note the greater retraction than in case A, with a larger gap in the tendon, torn tendon ends (below the arrows), and herniated (hyperechoic) fat in the lesion space (***).

All patients were preoperatively evaluated using clinical and ultrasonographic exams to gather preoperative data concerning the site and the type of lesion (Figure 1).

Patients’ anamnestic data were collected: age, BMI, works, previous injury, surgery, sport activity (if any), and previous therapies on the tendons.

### 2.3. Surgical Technique

All patients were placed in a prone position with feet protruding from the table and the tourniquet placed on the thigh. A posterolateral approach was performed in all cases to ensure a direct visualization of the sural nerve. The two ends of the lesion were armed using Vicryl 2 (Ethicon Inc; Johnson & Johnson, Somerville, NJ, USA) to perform Krackow suturing [16] each on side of the lesion. These two sutures were knotted when the foot was placed in plantar flexion position in order to connect the torn ends and maintain the tendon at an appropriate tension. Finally, reinforcement suturing was performed with a simple circumferential running suture using Vicryl 2-0 (Ethicon Inc; Johnson & Johnson, Somerville, NJ, USA). The limb was bandaged at 20° in equinus position with vascular elastic bandages. Furthermore, we intraoperatively recorded plantar tendon ruptures and insertional calcaneus deformities, such as Haglund’s disease.

### 2.4. Rehabilitation Protocols

In the following Table 2 and Table 3, the two rehabilitation protocols are reported in detail.

### 2.5. Postoperative Assessment and Study Outcomes

All patients were clinically evaluated 12 months after surgery. The clinical parameters used to test the degree of sural triceps hypotrophy were: calf circumference (4 cm below the anterior tibial tuberosity) and the tibial length (measured from the anterior tibial tuberosity to the medial malleolus). The American Orthopaedic Foot and Ankle Society (AOFAS) Ankle–Hindfoot Score [17] and The Achilles tendon Total Rupture Score (ATRS) [18] were used at the follow-up visits.

**Figure 2:**
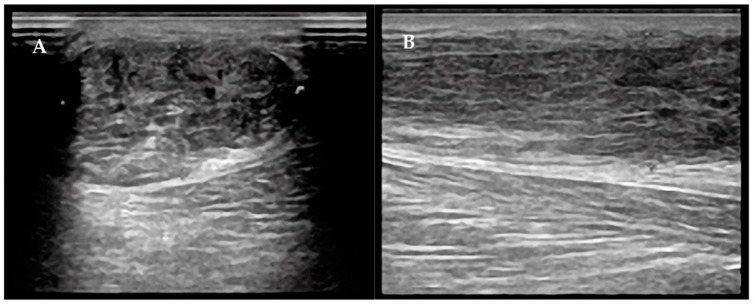
Transverse (**A**) and longitudinal (**B**) ultrasonographic images of the Achilles tendon after tenorrhaphy surgery following complete rupture. The loss of the fibrillar structure, the inhomogeneity, and the surgical material in the context of the tendon are "normal" aspects after surgical repair.

**Figure 3:**
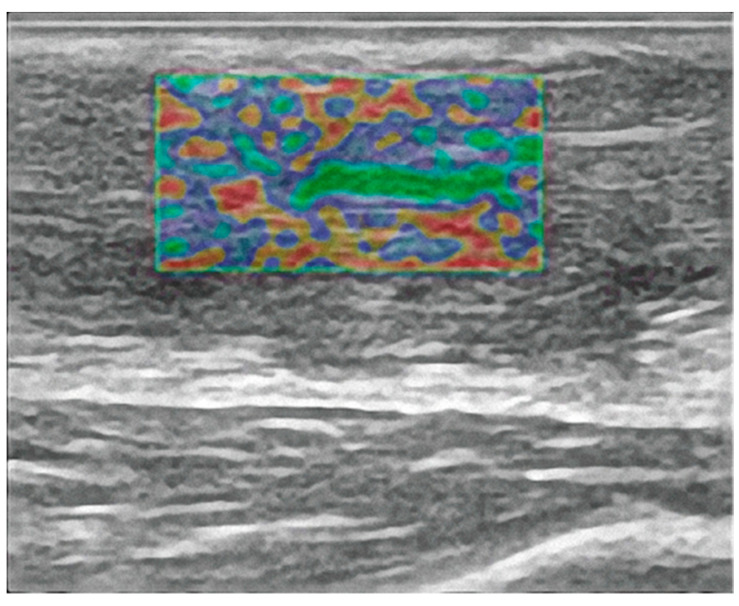
Longitudinal elastography image of Achilles tendon after surgery.

Furthermore, two different instrumental techniques were used in order to highlight the possible differences between the two operated tendons: ultrasonography (EsaoteMyLAb 70 ultrasound scanner with a 7.5 MHz linear transduce, Figure 2) and elastosonography (EsaoteMyLAb 70 with a 7.5 MHz linear transducer, using strain methodology, Figure 3), performed by a single radiologist. During ultrasonography (US), both the thickness of the tendon (ventrodorsal diameter) and the width (medial–lateral diameter) were calculated. These two measurements were collected along the tendon at three levels: proximal (musculotendinous junction), medial (2–6 cm above the insertion in the calcaneus), and distal (insertion at the heel).

Regarding elastosonography (ES), using the B-mode ultrasound display as a guide, three regions of interest (ROI) were selected for the same point at which ultrasound measurements were taken. In these regions, the level of red color on the color scale, which represents the elasticity values, was evaluated in order to assess tendon stiffness at the myotendinous level, at the medial third (the region of the rupture in the operated tendon), and at the distal level in the calcaneal insertion region.

### 2.6. Statistical Analysis

Continuous variables are presented as median and 25° and 75° percentile (IQR). Categorical variables are presented as frequency and percentages. To analyze the differences between groups, Mann–Whitney tests were applied for continuous outcomes, while Chi-square tests or Fisher’s tests were used for categorical outcomes. For all tests, a *p*-value less than 0.05 was considered statistically significant. All statistical analyses were performed using Stata (Stata Corp., College Station, TX, USA).

## 3. Results

Of the 33 patients that underwent open AT tenorrhaphy, 15 patients were treated using the fast functional rehabilitation program (group A), and 18 patients, with similar features in terms of clinical and demographic data, were treated with a plaster cast protocol (group B) (Table 4). Only one difference was found concerning the drug variable, as no one used drugs in group B.

In group A, the median waiting time between injury and surgery was 5.0 days (range 2–10 days); no intraoperative or postoperative complications were observed in this group.

In group B, the median waiting time between injury and surgery was 5.0 days (range 1–10 days).

Ultrasound and elastosonography were used to analyze the differences on the operated tendon between the two groups.

Comparing surgical side, no significant differences in ultrasonographic features were observed, similarly in thickness and length, in both groups (Table 5).

Comparing surgical side, no elastographic differences were observed in stiffness in both groups (Table 6).

Regarding the postoperative assessment scores, the results are overlapping, as shown in Table 7.

## 4. Discussion

Acute Achilles tendon injury is a common lesion, frequently occurring during sport activity, and its incidence seems to be increasing. This lesion can be treated conservatively or surgically, requiring, in any case, long periods of rehabilitation (not less than 6 months), with a permanent strength deficit of 10% to 30% [19]. Because of conclusive evidence that outcomes after surgical and nonsurgical treatment of Achilles tendon rupture are comparable [20,21,22], methods of rehabilitation are becoming increasingly significant. Traditionally, surgical management of acute Achilles tendon rupture was followed by a prolonged period of ankle immobilization in a rigid cast, which, on the other side, posed the problem of stiffness (due to adhesion and muscle atrophy). In order to limit the negative effects of prolonged immobilization, another postoperative protocol started to gain increasing popularity, based on early mobilization and tendon loading. This treatment, however, found a practical limit in the fact that its internal safety was not known, thus exposing the newly repaired tendon to stress that could cause its rupture.

Only when the safety of an early rehabilitation program was confirmed [23,24] did we begin to look with interest at a comparison between the traditional method and the functional postoperative rehabilitation.

In 2015, McCormack confirmed not only that dynamic functional rehabilitation, including weightbearing initiated within first two weeks, is safe, but presents higher patient satisfaction and earlier return to activity [15]. Many authors [20,25,26,27] suggest that fast rehabilitation programs after open Achilles tenorrhaphy help to accelerate and facilitate the physiological repair process of the tendon suture, encouraging faster recovery of daily life activities, and a return to preinjury sports performance. According to Huang et al. [28], faster mobilization of the ankle joint produces a better restoration of the articular range of motion, with an improvement in local edema, and prevents stiffness and calf atrophy [29]. In addition, in previous literature [30], early weightbearing and faster recovery of ankle range of motion is linked to a reduction in some complications, such as scarring adhesions, abnormal sensation, and lower risk of deep vein thrombosis.

Regarding clinical and functional outcomes, Ryu et al. [31] reported a case series of 112 patients treated with early rehabilitation for acute tear of the AT. All patients included in the study were fully satisfied, with an AOFAS at 1-year follow-up of 95.7, similar to our results (90 and 92). In a previous study, Kim et al. [32] compared the functional outcomes of patients treated with an early rehabilitation protocol and patients treated with cast immobilization. The AOFAS was slightly higher in the earlier group (93, compared to 89 of the cast group), and he reported a statistically significant difference between the two groups (*p* < 0.05).

Unlike the results McCormack and other colleagues reported, our results show no statistically significant differences in the analyzed outcomes between patients treated with the traditional plaster cast and the accelerated fast functional protocol at the twelfth month, despite the fact that our early functional rehabilitation protocol is in line with the previous literature data, including a variety of different exercise-based interventions. Active ankle mobilization was the most commonly included intervention [19], followed by progressive isometric strengthening of the sural triceps.

Aufwerber et al. [25] demonstrated that an accelerated postoperative protocol with immediate loading and ankle motion resulted in better general health and vitality at 6 months; however, the results at 12 months showing no significant differences in functional outcomes between patients in the early functional mobilization and control groups. This can probably be attributed to the minor differences between the treatment groups in terms of weightbearing status. These data are in line with our results, which, as already said, report a clinical and instrumental evaluation of the patient 12 months after surgery, not allowing evaluation of the patient during the first postoperative year.

This limit of our study, however, allows us to appreciate how, in the case of a long-term follow-up, the differences between the two rehabilitation treatments tend to smooth out. In our opinion, this represents further proof that all treatment, including the rehabilitation process, must be adapted and chosen according to the type of patient concerned. Thus, if we are dealing with young patients who require a rapid recovery of functional skills, we will opt for a fast rehabilitation protocol. On the contrary, in the case of a patient with fewer functional demands and lower compliance, the traditional cast treatment remains a valid postoperative option, smoothing out, in the first post-treatment year, the differences between the two groups of patients.

Methods of rehabilitation are becoming increasingly significant [20,33,34,35], but there is still limited available evidence for optimized rehabilitation regimen, limited guidelines for the initial rehabilitation [36,37], and limited data on the course of the recovery after Achilles tendon rupture and long-term outcomes of athletes [38], which are the ideal populations to treat using the functional protocol, always remembering that the compliance of patients is mandatory in this type of protocol. Since there is not a universally accepted protocol of early rehabilitation and timing of weightbearing, further studies are needed in the future [39]. The outcomes of the physiotherapeutic protocol could be influenced by the association with other treatments, such as high energy laser therapy or extracorporeal shock wave therapy, which are proven to decrease inflammation, decrease pain, and increase fibroblast activity, leading to increased collagen production and tensile strength [40,41].

From the anatomopathological point of view, when a repaired tendon is subjected to tension during healing, orientation of collagen fibers, strength of the calf muscles, breaking strength of the tendon, number of collagen filaments, and tendon vascularity are all improved [26]. In addition, the use of supplements and/or drugs (e.g., palmitoylethanolamide, triamcinolone acetonide, or Curcuma) can modulate the human-tendon-derived stem cell activity with potential implications on the tendon healing process [42]. We know from the literature that repaired tendons show different characteristics from healthy tendons, reaching a plateau by about 6 months postoperation. Bleakney and colleagues show that the repaired tendons are larger, wider, or both than unoperated tendons [43].

This progressive increase in size is observed during the first 3 to 6 months after rupture, and the extent of the increase may depend on the surgical technique adopted [44]. The augmented size could be due to a remodeling phase, which lasts several months after surgical repair, attributable to the production, deposition, and orientation of cross-linking fibrillar collagen [45]. In accordance with the literature, we found an increased general width (proximal, medial, and distal) in patients who underwent accelerated rehabilitation program compared to those treated with cast immobilization, with a reduced proximal and medial thickness, probably due to the remodeling phase. It should also be remembered that our data refer to a follow-up time of 12 months after surgical treatment, when the remodeling process, during which there is an increase in the size of the tendon, could have come to an end, explaining the presence of dimensional variations, both in one sense and the other, but also the lack of statistical significance, also probably due to the limited number of patients enrolled in the study.

Pathological processes at the level of the AT, as for many other tissues, modify its physical characteristics of elasticity, viscoelasticity, and mechanics. ES has been demonstrated to be a quick, relatively inexpensive, safe examination and, therefore, can be performed over time as many times as necessary without subjecting the patient to radiation. After surgery, we observed decreased stiffness in the medial third in the functional rehabilitation group compared to the group treated with cast immobilization. However, no statistically significant difference was found between the two groups.

The limitations of this study are the small sample size and the lack of protocol for patient randomization and double-blind trials. These last two points could have influenced the homogeneity of the patients assigned to the two clinical rehabilitation treatments (cast immobilization protocol versus accelerated protocol), or introduced potential prejudices in favor of one treatment over the other. Another limitation of the study, as already evidenced, is the lack of an intermediate follow-up that allows one to evaluate the clinical and instrumental status of the patient in the perioperative period.

## 5. Conclusions

Although no major statistical differences were reported in the clinical, US, and ES evaluations, our clinical results, according to the evidence reported in previous literature, confirmed that a fast rehabilitation after AT surgical repair is safe, effective, and may be the first choice of treatment, especially in young, collaborative, and active patients. Further studies should assess the contribution of energy-based treatments to the rehabilitation protocol.

## Figures and Tables

**Table 1:** Inclusion and exclusion criteria.

**Inclusion Criteria**
Patients ≤ 65 years old Primary lesion without previous physical or surgical therapy Medial third lesion Surgery within 10 days after injury
**Exclusion Criteria**
Patients ≥ 65 years old Recurrent lesion with previous physical or surgical therapy Medical conditions associated with higher risk of postoperative complications (rheumatic disease or collagenopathies) Third proximal or distal lesion Delayed surgery more than 10 days after injury

**Table 2:** Fast rehabilitation protocol applied to group A.

Fast Rehabilitation Protocol (Group A)	
0–7 days	Bandage at 20° in equinus position with elastic vascular bandages, passive, and active mobilization from 90° of dorsiflexion to maximum plantar flexion, walking with crutches out of charge on the operated limb.
7–45 days	Replace bandage with orthotic device during the weightbearing process, a plastic boot-shaped brace that aims to block the ankle movement in dorsiflexion, allowing the healing of surgical sutures. 1 cm wedges are placed inside the brace, depending on muscular and tendon tension reached after surgery. Every 10 days, one wedge is removed. During this period, a progressive increase in weightbearing is required. The brace has to be removed several times a day to mobilize the ankle.
>45 days	Progressive abandonment of the brace and starting isometric strengthening of sural triceps.
>60 days	Concentric strengthening of sural triceps.
>12 weeks	Stretching of sural triceps and gradual resumption of the race if the recovery of ROM of the ankle and muscle trophism is complete.

**Table 3:** Rehabilitation protocol with cast immobilization applied to group B.

Rehabilitation Protocol with Cast Immobilization (Group B)	
0–5 days	Plaster splint in equinus position until the first clinic control.
5–21 days	Immobilization in equinus cast, walking with crutches out of charge on the operated limb.
21–45 days	Replace equinus plaster splint with orthomorphic cast for 3–8 weeks (depending on surgical complications).
>45 days	Walking with crutches with partial weightbearing allowed, starting FKT of passive and active mobilization of the ankle, avoiding maximal dorsiflexion for two weeks.
>60 days	Isometric and concentric strengthening of sural triceps.
>75 days	Walking with complete load, starting Stanish exercises and eccentric strengthening.

**Table 4:** Patients features.

	Group B N = 18	Group A N = 15	*p*-Value
Age (years)	38.5 (34.0–44.0)	44.0 (39.0–49.0)	0.11
Sedentary job, n (%)	11 (61.1%)	7 (46.7%)	0.41
Sports, n (%)	12 (66.7%)	13 (86.7%)	0.18
Haglund, n (%)	18 (100%)	14 (93.3%)	0.46
Plantaris tendon rupture, n (%)	0 (0%)	1 (6.7%)	0.46
Flatfoot, n (%)	2 (11.1%)	1 (6.7%)	0.57
No pharmacological therapy, n (%)	18 (100.0%)	9 (60.0%)	<0.01
Calf circumference (cm)	37.0 (34.0–39.0)	36.0 (34.0–39.0)	0.60
Lesion on the right side, n (%)	12 (66.7%)	8 (53.3%)	0.44
BMI (cm)	24.9 (22.5–25.5)	23.2 (22.2–25.2)	0.25
Tibial length (cm)	35.0 (34.0–36.0)	36.0 (34.0–37.5)	0.19

Values are medians (IQR) or n (%).

**Table 5:** Ultrasound measurements on operated tendon; Mann–Whitney test (MW).

	Group B N = 18	Group A N = 15	*p*-Value
Proximal width (mm)	24.0 (20.0–26.5)	25.7 (18.8–30.1)	0.37
Proximal thickness (mm)	5.0 (3.6–6.5)	4.9 (2.8–7.0)	0.80
Medial width (mm)	24.3 (20.2–27.2)	26.3 (23.1–30.3)	0.19
Medial thickness (mm)	13.2 (11.6–15.0)	11.6 (8.7–16.9)	0.42
Distal width (mm)	19.3 (18.3–20.6)	21.3 (17.7–24.7)	0.12
Distal thickness (mm)	9.5 (7.8–12.1)	11.0 (7.8–16.6)	0.13

Values are medians (IQR).

**Table 6:** Elastosonography measurements on operated tendon; Mann–Whitney test (MW).

	Group B N = 18	Group A N = 15	*p*-Value
Proximal	90.0% (90.0–100.0%)	90.0% (90.0–100.0%)	0.74
Medial	80.0% (80.0–90.0%)	75.0% (70.0–95.0%)	0.18
Distal	80.0% (70.0–90.0%)	80.0% (60.0–80.0%)	0.54

Values are medians (IQR).

**Table 7:** Evaluation of postoperative recovery with ATRS and AOFAS; Mann–Whitney test (MW).

	Group B N = 18	Group A N = 15	*p*-Value
ATRS	87.5 (83.0–92.0)	84.0 (78.0–97.0)	0.70
AOFAS	92.0 (87.0–100.0)	90.0 (85.0–100.0)	0.97

Values are medians (IQR).
